# Identify successful restrictions in suppressing the early outbreak of COVID-19 in Arizona, United States: Interrupted time series analysis

**DOI:** 10.1371/journal.pone.0291205

**Published:** 2023-11-27

**Authors:** Ali Hadianfar, Milad Delavary, Martin Lavallière, Amir Nejatian, Omid Mehrpour

**Affiliations:** 1 Student Research Committee, Department of Biostatistics, School of Health, Mashhad University of Medical Sciences, Mashhad, Iran; 2 Department of Health Sciences, Laboratoire BioNR and Centre Intersectoriel en Santé Durable (CISD), Université du Québec à Chicoutimi, Chicoutimi, Québec, Canada; 3 Department of Civil Engineering, Sharif University of Technology, Tehran, Iran; 4 Arizona Poison & Drug Information Center, College of Pharmacy, The University of Arizona, Tucson, Arizona, United States of America; 5 College of Medicine, University of Arizona, Tucson, Arizona, United States of America; Fiji National University, FIJI

## Abstract

COVID-19 was responsible for many deaths and economic losses around the globe since its first case report. Governments implemented a variety of policies to combat the pandemic in order to protect their citizens and save lives. Early in 2020, the first cases were reported in Arizona State and continued to rise until the discovery of the vaccine in 2021. A variety of strategies and interventions to stop or decelerate the spread of the pandemic has been considered. It is recommended to define which strategy was successful for disease propagation prevention and could be used in further similar situations. This study aimed to evaluate the effect of people’s contact interventions strategies which were implemented in Arizona State and their effect on reducing the daily new COVID-19 cases and deaths. Their effect on daily COVID-19 cases and deaths were evaluated using an interrupted time series analysis during the pandemic’s first peaks to better understand the onward situation. Canceling the order of staying at home (95% CI, 1718.52 to 6218.79; *p*<0.001) and expiring large gatherings (95% CI, 1984.99 to 7060.26; p<0.001) on June 30 and August 17, 2020, respectively, had a significant effect on the pandemic, leading to the daily cases to grow rapidly. Moreover, canceling the stay at home orders led to an increase in the number of COVID-19 daily deaths by 67.68 cases (95% CI, 27.96 to 107.40; p<0.001) after about 21 days while prohibiting large gatherings significantly decreased 66.76 (95% CI: 20.56 to 112.96; p = 0.004) the number of daily deaths with about 21 days’ lag. The results showed that strategies aimed at reducing people’s contact with one another could successfully help fight the pandemic. Findings from this study provide important evidence to support state-level policies that require observance of social distancing by the general public for future pandemics.

## Introduction

Respiratory droplets have been recognized as the primary mode of transmission for COVID-19, which is highly contagious and similar to influenza virus transmission [1, 2]. This makes disease propagation faster especially in closed spaces where people are at higher odds of proximity and contact [3].

On January 20, 2020, the United States had its first confirmed case of COVID-19 disease [4]. By the end of 2020, one-fourth of Americans were infected due to the rapid spread of the SARS-CoV2 virus throughout the country. No vaccine or treatment had been approved for the disease until late 2020 [5], making the situation more difficult for Americans. The COVID-19 reproduction number is reported to be about 1.90 to 6.49, which means each infected person can infect about two to six other individuals [38, 39] causing the disease to spread fast. In this situation if the transmission routes are not blocked, the disease may propagate exponentially and produce high peaks in daily case reports and deaths [40]. The government’s attention was drawn to what could be done before vaccines were discovered in the event of pandemic. Experts have suggested different policies based on the disease’s major transmission routes. Social distancing and mask-wearing were the most commonly suggested procedures to slow down the COVID-19 progression [6, 7].

Each country implemented different policies to suppress the pandemic like closing schools and universities in Iran, Zambia [8–10], prohibiting restaurant dine-in services in US and Brazil [11–13], closing gyms in Saudi Arabia and US [14, 15], ban travelers in Europe and US [16–19] and total or partial lockdowns to completely prevent any contact between people in France, Germany, Italy, Russia, Denmark and many other countries [20, 21]. Furthermore, implementing some strategies has economical and mental costs [22, 23]. For instance, closing gyms and restaurants or canceling holidays tours directly affected people’s revenue and markets [24], or closing schools and holding online classes impacted students’ learning [10]. Thus, each policy must be scrutinized to determine if it cease or interrupted the pandemic or just caused potential financial or psychological losses to people. This can lead to sieve suitable strategies and cancel the others when such a pandemic occurs again, as evidenced by previous outbreaks in Africa caused by different viruses.

Researchers studied the effects of different policies on peoples’ behavior or mental situation [22, 23], markets [24], and agriculture [25], but their effect on the pandemic is not clear. There is also a need to investigate the effect of policies for each country or state individually, since people’s culture, beliefs, and also political views make them respond differently to implemented policies and therefore change the study outcome of each policy’s effectiveness [26–29]. In this case, the center for disease control and prevention (CDC) has gathered preventive strategies information for each state of the United States (US) and their stringency as an exploratory tool but did not determine their effect on reducing daily positive cases or deaths in a scientific way [30]. Therefore, there is a significant need to scientifically determine whether a policy was successful in reducing COVID-19 cases or deaths in a society or requires further considerations. To fill in this important gap this study aims to determine which of the policies undertaken in Arizona State in the US were beneficial in suppressing the pandemic spread and could be used in the COVID-19 next variants or in similar pandemic situations in the future.

## Materials and methods

### Case study

The current study has been conducted in Arizona, United States, whose population is 7.27 million and which has arid and semi-arid climate [31]. The daily number of COVID-19 cases and deaths from March 11, 2020, to the end of 2020 was taken from the Arizona Department of Health Services (ADHS) dashboard [32] and the Centers for Disease Control and Prevention (CDC) respectively [33]. Summary of statistics including mean, standard deviation, minimum, maximum, skewness and kurtosis are presented in Table 1.

**Table 1:** Descriptive statistics of daily new cases of COVID-19 cases and deaths in Arizona State from March 11, 2020, to December 31, 2020.

Statistics	New cases	New deaths
Mean	1941.1	30.2
Std. dev	2362.5	35.2
Min	4.0	0.0
Max	11748.0	172.0
Skewness	1.8	1.8
Kurtosis	3.0	3.4

According to the Office of the Governor’s website, Arizona has taken a variety of measures to stop the spread of COVID-19. Policies aimed cutting people’s contact were used to affect disease transmission. As of March 11, 2020, there have been no confirmed COVID-19 cases in the state of Arizona. Consideration was given to the following policy initiatives: The first policy was the *closing bars*, *gyms*, *and movie theaters*, implemented from March 19, 2020 to May 16, 2020. The second policy was the *stay-at-home executive order*, *stay healthy*, *and stay connected* from March 30, 2020 to May 16, 2020. The third intervention was *canceling or postponing the order to stay at home*, implemented from May 16, 2020, to June 30, 2020. Finally, on June 30, 2020, a new legislation came into force with the *order to prohibit large gatherings* from being held and it continued till August 17, 2020.

### Statistical analysis

Using SARIMA modeling, this study examined the impact of interventions, which are one-time events that have an impact on new confirmed cases. The Dynamic Regression Model, also known as dynamic regression, was first introduced by Box and Tiao as a framework [34]. Although regulations and law enforcements are implemented in a specific period, its impact cannot be observed immediately. The current study utilized two types of intervention, including one-time decrease or increase and decayed (exponential trend) response, to evaluate the governmental policies. The standard interrupted time series regression model is as follows:

$$Y_{t}=\beta_{0}+\beta_{1}X_{1t}+\;\beta_{2}X_{2t}\;+\beta_{3}X_{3t}+\beta_{4}X_{4t}+e_{t},$$ (1)

Y_t_ is the response variable counting the number of new COVID-19 cases or deaths during the studied period. *β*_0_ shows the intercept of model and, *β*_1_, *β*_2_, *β*_3_, and *β*_4_ indicate the impact of the changes in the new COVID-19 cases or deaths due to the closing of bars, stay-at-home order, canceling the stay-at-home order, and prohibiting large gatherings, respectively, with the statistical significance level (p = .05). It is worth mentioning that SARIMA modeling was used to capture the error term. To confirm the white noise of residual, Ljung–Box test was used to check the uncorrelation [34]. Also, zero-mean and normal distribution of residuals was investigated using residual plots and Kolmogorov-Smirnov test, respectively. All statistical modeling was done in R4.0.0 using a *forecast* package [35].

### Ethical statement

The data is publicly available online on the ADHS website [https://www.azdhs.gov/covid19/data/index.php#confirmed-by-day] and CDC website [https://covid.cdc.gov/covid-data-tracker/#datatracker-home]. Therefore, ethical approval was not required.

## Results

**Fig 1:**
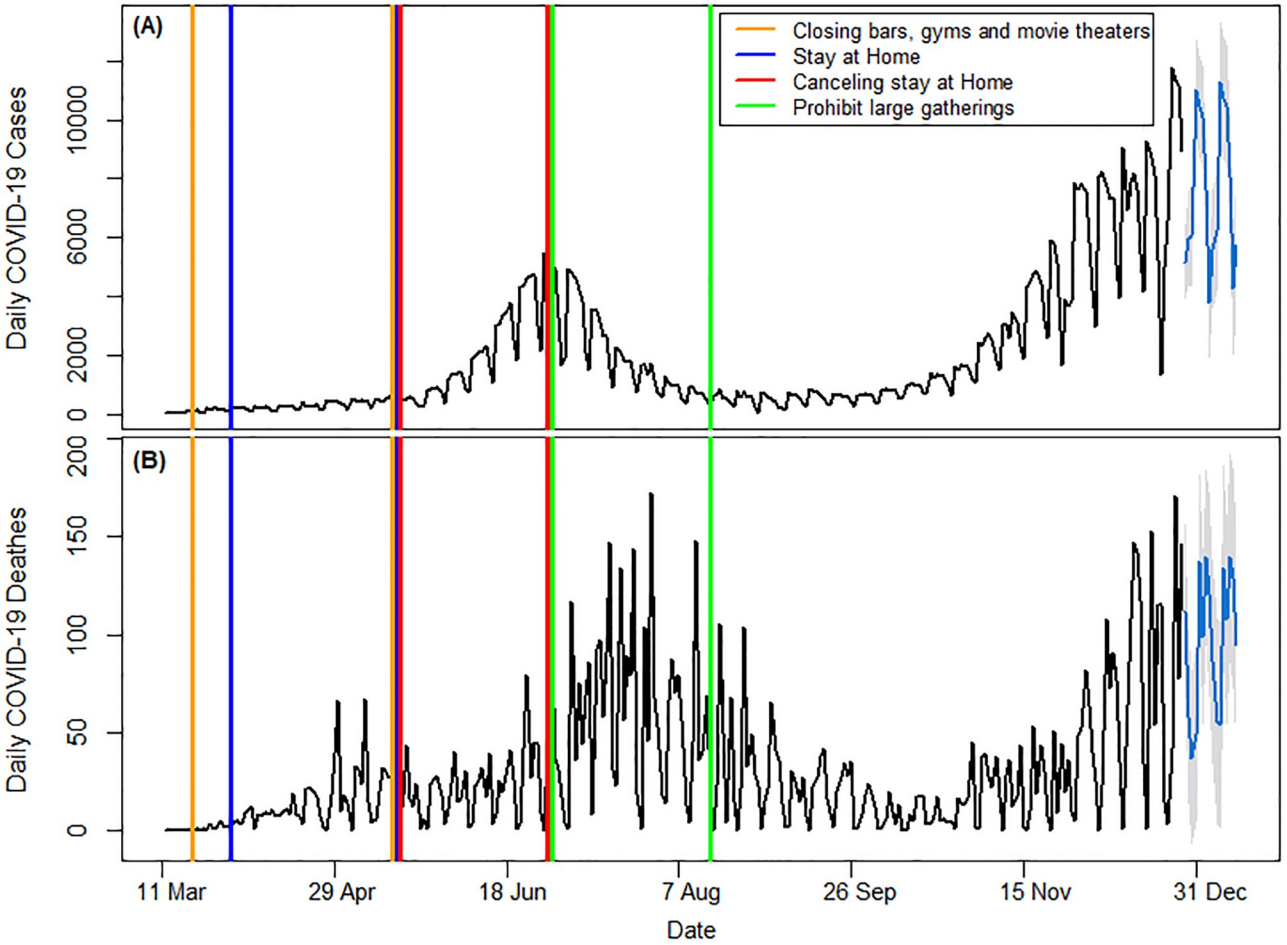
The Time Series of daily New Confirmed COVID-19 Cases (A) and daily New COVID-19 deaths (B) in the Arizona State (USA) From March 11, 2020, to December 31, 2020, and Implemented Interventions.

The Fig 1 illustrates the time series of daily COVID-19 cases and daily COVID-19 deaths in Arizona State, along with their corresponding forecasting values. Specifically, the orange and blue lines indicate bar closures, gym closures, and movie theater closures. There are red lines representing cancellations of orders (which increased significantly), and green lines representing prohibitions of large gatherings (which decreased significantly).

The first line graph (A) in this figure portrays how the number of new confirmed COVID-19 cases in Arizona gradually escalated from 4 cases on the first day of the outbreak to 5450 cases on June 29, 2020. It then gradually subsided, reaching its lowest point of 23 cases on August 30, 2020. Thereafter, until October 4, 2020, the daily new confirmed cases showed a monotonic time series. However, towards the end of December 31, 2020, the time series will have an increasing trend with high variation.

The second line graph (B) illustrates the daily number of COVID-19 deaths in Arizona State for the year 2020. The trend is comparable to the daily new cases trend, yet displays more fluctuation. The first COVID-19 death in Arizona State was recorded on March 20, 2020, which was followed by a surge in deaths, peaking on July 30. Although the number of deaths gradually decreased until October 11, there were still high fluctuations towards the end of December 31, 2020.

As shown in Table 2, the results of the daily new COVID-19 cases model show that closing bars, gyms, and movie theaters and the stay-at-home order had no significant impact on the new COVID-19 cases (p>0.05). However, the implementation of canceling of the stay-at-home orders as the third intervention led to an increase in the number of new confirmed COVID-19 daily cases by 3968.66 cases (95% CI, 1718.52 to 6218.79; *p*<0.001) from May 16, 2020 to June 30, 2020. Thereafter, the ordering to prohibit large gatherings on August 17, 2020 significantly decreased the number of new confirmed COVID-19 cases by 4522.63 (95% CI, 1984.99 to 7060.26; *p*<0.001).

**Table 2:** The effects of the government interventions on the COVID-19 daily new cases and deaths in Arizona State, SARIMA models.

Model	Output	Estimate	SE	P-value	95%CI	Ljung–Box	Kolmogorov–Smirnov
**New Cases**	**Intervention 1** ^a^	52.90	390.88	0.89	(-713.22, 819.02)		
	**Intervention 2** ^b^	23.14	380.97	0.95	(-723.56, 769.84)		
	**Intervention 3** ^c^	3968.66	1148.03	<0.001	(1718.52, 6218.79)		
	**Intervention 4** ^d^	4522.63	1294.71	<0.001	(1984.99, 7060.26)		
	**Noise**	(2,0,0) (1,1,0)_7_				0.07	0.72
**New deaths**	**Intervention 1** ^a^	-0.25	8.35	0.97	(-16.62, 16.13)		
	**Intervention 2** ^b^	2.36	8.23	0.77	(-13.76, 18.49)		
	**Intervention 3** ^c^	67.68	20.26	<0.001	(27.96, 107.40)		
	**Intervention 4** ^d^	66.76	23.57	0.004	(20.56, 112.96)		
	**Noise**	(0,1,1) (0,1,2)_7_				0.12	0.96

^a^ Closing bars, gyms, and movie theaters

^b^ Order to stay at home, stay healthy, stay connected

^c^ Canceling or postponing order to stay at home

^d^ Order to prohibit large gatherings

The error term for this model is *SARIMA*(2.0.0)(1.1.0)_7_, which shows that there is a recurrent pattern (seasonal term) of change in number of new daily cases of COVID-19 in Arizona State. This seasonal effect is seven days, showing a uniform pattern that happens every one week.

As a mandatory criterion for accepting the model, the residuals should be white noise for diagnostics. In this regard, the residuals plot shows no pattern and is randomly distributed around zero. Furthermore, there are no spikes in the autocorrelation function, indicating that there is no residual autocorrelation. Uncorrelated residuals were confirmed, (p-value = 0.07), using the Ljung-Box (LB) test for the time series at the 5% significance level, as shown in Table 1. Furthermore, the Kolmogorov-Smirnov (KS) test confirmed the residuals’ normality (p-value = 0.72).

The results of the daily COVID-19 deaths model indicate that closing bars, gyms, and movie theaters and the stay-at-home order had no significant effect on the COVID -19 deaths (p-value>0.05). However, canceling the stay-at-home order, as the third intervention led to an exponential increase of 67.68 COVID-19 deaths per day (95% CI, 27.96 to 107.40; p<0.001) after about 21 days. The study found that implementing the Order to prohibit large gatherings led to a significant decline of 66.76 (95% CI: 20.56 to 112.96; p = 0.004) COVID-19 deaths per day after about 21 days, as evidenced by a positive coefficient for a decayed exponential effect. This lagged-effect might be related to the incubation period of the disease, which has been reported in various epidemiological studies [36, 37].

The error term for this model is *SARIMA*(0.1.1)(0.1.2)_7_, which indicates a weekly seasonal pattern in the daily number of COVID-19 deaths in Arizona State. This season is seven days, which shows that a uniform pattern that occurs at regular intervals of one week.

In this model also, the hypothesis of the un-correlation and the hypothesis of normality of the residuals were accepted by LB and KS tests respectively (p-value>0.05).

## Discussion

Arizona’s first COVID-19 cases were reported in early March 2020 and slowly reached its first peak on June 29. Since the disease was highly contagious and no cure was available for COVID-19, the best and only solution was to cut the contact between the healthy and ill [38]. Hence, bars, gyms, and movie theaters were ordered to close on March 19, 2020, and the executive order on March 30, 2020 requested people to stay at home. Although lockdowns generated economic stress on people and society, they were implemented in different countries worldwide since they were considered the best way to reduce peoples’ contact with infected individuals or surfaces [20, 21]. Studies further confirmed lockdowns and other strategies can significantly impact the pandemic growth by cutting the contact between healthy and ill individuals [20, 39]. These two interventions, implemented at the beginning of the pandemic in Arizona State, did not significantly show their effect on cutting the transmission route, as the disease was still spreading slowly. People who were asymptomatic still carried and transmitted the SARS-CoV2 virus within society, leading to more deaths and financial losses [40, 41]. The results of this study indicated that closing bars, gyms, and movie theaters and ordering people to stay at home did not have a significant effect on the spread of the pandemic or death numbers. However, as illustrated by the first line graph (A) in Fig 1, the line chart between the yellow lines is monotonic, indicating that these two policies effectively halted the spread of the pandemic. They also preserved death numbers in a horizontal channel which means although they could not effectively decrease death numbers, they maintained their rate and prevented them from increasing, as it is shown in the second line graph (B) in Fig 1.

It may seem that the stay-at-home order and closing populated and contagious places did not sufficiently reduce contact between healthy and ill individuals, as the disease continued to spread slowly. As a result, the stay-at-home order was canceled on May 16, 2020. However, the cancellation of the order revealed its impact later on, as the situation became more severe and confirmed cases of COVID-19 rapidly increased. The pandemic started to spread rapidly after the stay-at-home order was lifted and peaked in about 45 days later, highlighting the importance of lockdowns and their effectiveness in reducing disease transmission. Lockdowns were also implemented in various countries, and research across 49 countries indicated that the spread of the SARS-CoV2 virus was significantly reduced during lockdowns [20]. Canceling the stay at home order not only led to a peak in new daily cases but also created a peak in death numbers, which was observed with a 21-day lag. The increase in death numbers are also came with a lag, as shown in the second line graph (B) in Fig 1. This is due to the disease growth stages, which can lead to death in addition to the disease incubation period of about 7 to 14 days. It takes time for infected individuals to progress from the first day of the disease to eventually succumbing to the illness, as observed by other studies which have reported a 21-day lag between new COVID-19 cases and death numbers [42, 43]. A 10 to 14 days lag has also been observed between state-level policy decisions and their impact on changes in COVID-19 outcomes [44]. This is also important for Arizona State policy makers to consider that implemented policy for controlling COVID-19 showed its effect on death numbers after 21 days. They must make faster decisions on what needs to be done in future similar situations. The result of this study can be of great help in determining which policies can better cease similar pandemics in the future. The daily COVID-19 positive cases started to decrease after their first peak on June 29, 2020, following the large gathering prohibition order but started to increase significantly after two months, right after its cancelation on August 17, 2020. The peak in new daily cases in the last days of June caused a peak in death number in the last days of July. However, the large gathering prohibition, which successfully reduced daily cases, also had a positive impact on daily deaths and showed its effect with a 21-day lag. This is in agreement with another study showing that social distancing implemented in 28 European countries decreased daily COVID-19 cases growth rate from 24% to less than 2% [39]. A research on the social distancing order in Iran revealed that it could significantly decrease new cases by an average of 2128 per day [8]. How the strategy is implemented and how strictly it is enforced is more important than the specific strategy chosen to combat the pandemic. Determining this requires separate measurements, examinations, and field observations. For example, during the lockdown period in Wuhan, China, airports, highways, train stations, and most businesses were closed, and people were instructed not to leave their homes except for essential medical needs [45]. Strict measures were taken, and even traffic cards were issued, allowing people to leave their homes once every two days for 30 minutes. Although this led to problems such as increased food prices and difficulties for doctors and patients to travel to hospitals, it was able to stop and reverse the growth of infected individuals, and the doubling rate of patients increased from two to four days [46]. However, during the stay-at-home order in 41 states in the United States, people could still go to parks or beaches and travel between cities and states. Even if parks were closed in some states, people could still meet each other outside of their homes, and public transportation was still active [45]. This implies that the lockdown was not implemented as it should have been and only caused financial loss to society. As a result, the stay-at-home order was revised, some businesses reopened, and people were asked to follow health directives, avoid gathering, and observe social distancing through the prohibition of large gathering intervention. Social distancing requires certain precautions and environmental conditions that even differ between streets and neighborhoods. For example, the width of sidewalks, the number of seats available in a park, and the density of the population in a given area, as well as the financial ability of residents, can all impact how well people can observe social distancing. Additionally, due to the novelty of the disease and its initial spread, people may not take health directives seriously and may not be aware of the new conditions and harms caused by the disease. This led to an increase in the number of patients after the stay-at-home order was canceled. However, after the implementation of the prohibition of large gatherings, along with more information about the new disease and observations of deaths in society, people’s attitudes towards warnings have improved. Implementation of this order has led to a decrease of 4522.63 in the daily number of patients. The prohibition of large gatherings achieved relatively better results compared to the lockdown. But a detailed comparison of the effects and levels of lockdown and the prohibition of large gatherings requires more data. What that is important is the cooperation of people with the strategies developed to deal with issues such as the COVID-19 pandemic and how strictly they are followed. This indicates that cutting people’s physical interactions when a highly contagious disease starts to spread can be considered very useful and effective. It is also possible to reduce the number of new cases on a daily basis by enforcing lockdowns of various types of lockdowns, including the prohibition of travel within and between cities [34]. However, these strategies may have adverse psychological and financial consequences. Consequently, other approaches that reduce contact while maintaining employment are highly recommended. For example, the implementation of mandatory mask use in New York City resulted in a decrease in the average number of daily positive cases from 8549 to 5085. Other countries, such as Germany [47], Australia [48], have also found success with mandatory masking, as has the American CDC, which has recommended it, along with many other countries, including China and Hong Kong [49]. These findings suggest that intercepting human contact can significantly affect the spread of pandemics. Other strategies, such as wearing masks or socially distancing oneself from a potential virus source, can also be used in conjunction with intercepting human contact policies. These findings cannot be extrapolated to other states, but they do serve as a benchmark for determining which safety strategy yields the best results. The effectiveness of government policies may be affected by a variety of factors, including the availability of healthcare, adherence to health regulations, and the availability of preventive programs.

## Conclusion

The time-series analysis of the early outbreak of COVID-19 in Arizona State, covering 300 days, showed that policies targeting people’s contact were useful and could help suppress disease spread and death occurrence. The two most successful interventions were stay-at-home order and prohibition of large gatherings. The cancelation of the stay-at-home order led to a significant increase in daily COVID-19 cases and the pandemic’s first peak, followed by a peak in death numbers. On the other hand, implementing the prohibition of large gatherings led to a significant decrease in daily new cases. These orders can be implemented in new contagious diseases or new variants of COVID-19 pandemics. Further studies can explore new ways to reduce contact while preserving the social and economic requirements of the city, such as developing remote apps, utilizing online calls and meetings, and using special robots.

## Supporting information

S1 Data (XLSX)
